# Associations of neighbourhood walkability with patterns of device-measured stepping, standing and sitting

**DOI:** 10.1186/s12966-025-01737-4

**Published:** 2025-04-09

**Authors:** Christian J. Brakenridge, Elisabeth A.H. Winkler, James F. Sallis, David W Dunstan, Neville Owen, Takemi Sugiyama, Manoj Chandrabose

**Affiliations:** 1 School of Health Sciences, Swinburne University of Technology, John Street Hawthorn, Melbourne, VIC 3122 Australia; 2 Active Life Lab, South-Eastern Finland University of Applied Sciences, Mikkeli, Finland; 3 School of Human Movement and Nutrition Sciences, The University of Queensland, Brisbane, QLD Australia; 4 Herbert Wertheim School of Public Health & Human Longevity Science, University of California, San Diego, USA; 5 Mary MacKillop Institute for Health Research, Australian Catholic University, Melbourne, Australia; 6 Baker Heart & Diabetes Institute, Melbourne, VIC Australia; 7 Institute for Physical Activity and Nutrition, School of Exercise and Nutrition Sciences, Deakin University, Melbourne, VIC Australia

**Keywords:** Built environment, Physical activity, Sedentary behaviour, Public health, Urban design

## Abstract

**Background:**

Neighbourhood walkability is known to be positively associated with self-reported and device-based measures of overall physical activity. However, relations of walkability with specific active and sedentary behaviour patterns are not well understood.

**Methods:**

We investigated cross-sectional associations of neighbourhood walkability with time spent stepping, standing, sitting, and their pattern metrics using data from 505 participants (mean age 59.2 years) from the AusDiab3 study. Neighbourhood walkability (a composite measure of residential, destination, and intersection densities) was calculated within 1 km street-network buffers around participants’ homes. Thigh-worn device data (activPAL, 7-day, 24 h/day protocol) were used to derive stepping, sitting and standing minutes per day and their pattern metrics. Two-level linear mixed models assessed relevant associations, adjusting for potential confounders.

**Results:**

Higher walkability was associated with higher cadences (β [95% CI] = 0.12 [0.04–0.20]), moderate-to-vigorous physical activity (β [95% CI] = 0.17 [0.09–0.26]), longer stepping bouts (β [95% CI] = 0.18 [0.10–0.25]) and time in purposeful (≥ 2 min duration) walking (β [95% CI] = 0.21 [0.13–0.30]). There were no associations with total sitting time, standing time, or their associated pattern metrics. Total stepping time also had no associations, suggesting that participants in neighbourhoods with higher walkability may accumulate similar levels of stepping time to participants in lower walkability neighbourhoods, albeit with higher intensity and in longer bouts.

**Conclusions:**

By examining activity totals only, relevant walkability relationships may be masked. Further research is needed to understand whether walkability and other built environment attributes are associated with sedentary behaviour patterns, as well as light-intensity physical activities.

**Supplementary Information:**

The online version contains supplementary material available at 10.1186/s12966-025-01737-4.

## Background

Neighbourhoods that are designed to support active living can benefit health at the population level [1]. Walkability is an important concept in this context, referring to the extent to which an area is conducive to walking, especially for transport purposes [2]. It is typically conceptualised as a composite measure of attributes of the built environment: dwelling density; street connectivity; land use mix; and net retail area [2]. Higher levels of neighbourhood walkability, characterised by well-connected streets and access to different types of destinations, can be associated with greater levels of walking, both for transportation [3] and recreation purposes [4], and with total physical activity [5]. However, much of the walkability and physical activity research has used self-report activity measures [1, 5, 6].

Investigating the associations of neighbourhood walkability with device-based physical activity measurements (such as using pedometers or accelerometers) can provide a more accurate understanding of these relationships, as they eliminate recall and response bias inherent in self-reported activity. There are studies that have examined the associations of walkability with device-measured physical activity, mostly using total moderate-to-vigorous physical activity (MVPA) time obtained from accelerometers [5] or pedometer-derived daily step counts [7]. Specifically, device-measured step counts can be a conceptually relevant outcome measure for walkability because they can accurately capture walking [8]. A review of neighbourhood walkability and steps in adults, published about a decade ago [7], identified only a limited number of studies, with positive associations reported in three studies [9–11] and no associations in two studies [12, 13]. Subsequent research has also reported mostly null associations between walkability and device-measured total step counts [14–16].

Sedentary behaviours (defined as sitting or reclined behaviours with low energy expenditure [17]), have detrimental associations with health that are distinct from too little moderate-vigorous physical activity [18]. Exploring the relationships of neighbourhood walkability with sedentary behaviour is also relevant, because areas with low walkability levels (i.e., low density, limited access to destinations, and less connected streets) can present barriers to walking for transportation and thus promote car use. For instance, an Australian study found that lower walkability was associated with greater self-reported time spent sitting in cars [19]. However, a review published in 2015 reported mixed findings for the associations of walkability with self-reported transport, leisure, and total sitting time [20]. For device-measured total sitting time, a New Zealand study found that neighbourhood walkability was not associated with total accelerometer-measured sitting time, but specific walkability components such as access to destinations and street connectivity were negatively associated with it [21]. Other studies have also reported that walkability was not associated with device-measured total sitting time [22, 23], and some have reported positive associations between them [24–26].

It is known that people accumulate physical activity and sedentary behaviour in diverse ways. For instance, one can accrue one hour of physical activity through many short bouts of or a few longer bouts of physical activity. Thus, lack of consistent associations between neighbourhood walkability and total amounts of stepping and sitting could mean that walkability is only related to patterns of behaviours. As these patterns can influence health independent of total time spent walking [27] or sitting [28, 29] they warrant investigation with neighbourhood walkability. For stepping, investigating cadence, intensity, and bout durations could be useful. It has been shown that brisk walking (i.e., faster stepping cadence) is more beneficial to health than slower stepping [30, 31]. Similarly, moderate-or-vigorous intensity physical activity may offer greater and distinct health benefits compared to light-intensity walking [31, 32]. Additionally, longer, uninterrupted bouts of stepping may have stronger beneficial associations with health [33]. For sedentary behaviour, prolonged (uninterrupted) sitting bouts have been shown to be detrimentally associated with cardiovascular disease [28], and more standing time is associated with beneficial metabolic outcomes [29]. Findings may also differ by population studied. For instance, in one study, older adults had weaker associations between neighbourhood walkability and physical activity [22], Another study reported women had stronger associations of neighbourhood walkability with MVPA than did men [34]. These findings suggest non-significant associations for the whole sample could hide significant relations for subsamples. It is therefore important to determine whether the associations of interest differ according to socio-demographic attributes. Age and sex are particularly relevant in this context, as they are known effect modifiers of the relations between the built environment and behaviours as shown in previous studies [34, 35].

To date, only one study has investigated associations of neighbourhood walkability with stepping and sitting patterns derived using a device capable of determining the activity cadence, intensity, bout length, and distinguishing sitting from standing [22]. However, this study targeted older adults and used a self-report walkability measure. Thus, evidence is not yet available on associations of device-based pattern metrics with objectively measured neighbourhood walkability. As studies have reported a mismatch between perceived and objective built environmental measures [36], such evidence is needed to obtain a more comprehensive understanding of the relationships of built environments with patterns of physical activity and sedentary behaviour.

The aim of the present study was to examine associations of neighbourhood walkability with device-measured stepping, sitting, and standing and pattern metrics: mean stepping cadence, moderate-to-vigorous physical activity, mean stepping bout duration, stepping in bouts longer than 2 min, and mean sitting bout duration. How age and sex moderated these relations were also explored.

## Methods

### Participants and setting

Data were from the third wave of the Australian Diabetes, Obesity and Lifestyle Study (AusDiab3) conducted as a cohort follow-up in 2011/2012. Detailed descriptions of study design, recruitment procedures, and measurement methods have been reported previously [37]. In brief, the baseline study in 1999–2000 was a national population-based survey of 11,247 adults aged ≥ 25 years (response rate: 55.3%). Eligible baseline participants were non-institutionalized adults with no physical or intellectual disabilities who had resided in private dwellings for at least six months prior to data collection. A two-stage stratified cluster sampling approach was used for recruitment, with participants selected from 42 randomly chosen sites across the six states and the Northern Territory of Australia (six sites per state/territory). Subsequent follow-ups were conducted in 2004–2005 (*n* = 6400) and 2011–2012 (*n* = 4614). At the 2011/2012 follow-up, a sub-sample of participants were invited to wear an activity monitor; detailed procedures described elsewhere [38]. Eligible participants who were ambulatory and not pregnant, were recruited to wear activity monitors (*n* = 1,014). A total of 782 consented to wear the monitor, with 726 providing at least four valid days of monitor wear. For current analyses, exclusions were applied to participants who had no information on precise residential addresses to calculate neighbourhood walkability (*n* = 22) and those who were living in outer-regional and remote areas (*n* = 199) because environmental attributes that promote physical activity in urban contexts (e.g., walkability components) are not generally applicable to rural areas [39]. For identifying those rural areas, we used the Australian Remoteness Area classification, defined by the Accessibility/Remoteness Index of Australia (ARIA+), which was derived in 2011 using census population size and distance to urban centres [40]. In total, 505 participants were included in analyses.

### Exposure measure: neighbourhood walkability

The exposure variable was neighbourhood walkability index, and details of calculating this for the AusDiab3 study participants have been described previously [41]. Briefly, for each participant, their neighbourhood was defined using a 1 km street-network buffer around their home [42], because it has been shown that most home-based walking activities occur within this distance [43, 44]. The measures of residential density, destinations density and street connectivity were used as the components of walkability. The 2011 Australian Census dwelling count data was used to calculate residential density (count of dwellings divided by the buffer area). For destination density, we used the number of regularly visited destinations (supermarkets, convenience stores, public transport stops) [45], which were obtained from Axiom Business Points (2013) and PSMA Australia’s 2012 Transport & Topographic datasets. Street connectivity was measured by the density of 4-or-more-way intersections using the PSMA 2012 Transport & Topographic dataset. We used ArcGIS v.10.6 (ESRI Inc, Redlands, California) to calculate these built environmental attributes. The walkability index was calculated as the standardised score of the summed z-scores of residential density, destinations density, and street connectivity.

### Outcomes: device-measured stepping, standing, sitting and associated pattern metrics

Device derived activity data were recorded by the activPAL3 monitor (PAL technologies Limited, Glasgow, UK; version 6.4.1). This device has demonstrated accuracy and reliability in adults and older adults [46]. At the assessment, participants were instructed to wear the device for seven consecutive days and use a diary to record their sleep and wake times each day, as well as any removals of the device. An invalid day was defined when monitor wear time was less than 80% of waking hours, or less than 10 h if the participant’s diary was missing sleep and wake times. In the instance where sleep and wake times were not reported, an automated algorithm was applied to determine sleep and wake times, as per previous methods [47]. Upon completing wear, participants mailed back their monitors by post. Monitor data were then processed using a bespoke SAS 9.3 (SAS Institute Inc., Cary, NC, USA) program which derives average valid day summaries of each activity and pattern metric from activPAL events files. All outcomes with total minutes per day were standardised to 16-hours waking period to account for varying participant sleep and non-wear periods. Table 1 shows the activPAL-derived outcome variables, calculated per participant and averaged across all valid days.

**Table 1:** Outcome variables

Outcome variable (unit)	Method used (for activPAL device)	Rationale for inclusion and/or relevance to health
Stepping time (mins / 16 h day)	Time spent stepping. Standardised to 16 h awake and wearing the device.	Stepping time includes both dynamic light activity and moderate-vigorous intensity activity, and forms much of total physical activity time during the day. All of these have been associated with health outcomes.
Mean stepping cadence (steps / min)	Number of steps divided by time spent stepping.	Continuous measure of faster stepping speed (cadence) that has been positively associated with health outcomes and tends to indicate higher activity intensity.
Moderate-to-vigorous physical activity time (MVPA; mins / 16 h day)	Time stepping with cadence of ≥ 100 steps/min. Standardised to 16 h awake and wearing the device. Validated as a measure of moderate-to-vigorous intensity physical activity [72]	Benefits attained from physical activity may be optimised by increasing the intensity above a certain threshold. Time spent at or above the MVPA threshold are associated with distinct physiological processes and health benefits.
Mean stepping bout duration (mins)	Total stepping time divided by the number of stepping bouts.	A continuous pattern indicator of how long at a time people step without stopping. Longer versus shorter periods of being active continuously may reflect different behaviours and may have different relationships with health.
Purposeful walking time (mins / 16 h day)	Time spent stepping continuously for ≥ 2 min. Standardised to 16 h awake and wearing the device.	Purposeful walking time is a subset of total stepping time that may be particularly relevant for walkability, as it refers to time stepping from one place to another. Previous research [59] has tentatively identified a threshold of ≥ 2 min continuous stepping as separating purposeful walking from incidental stepping (where steps occur interspersed among other activities such as standing).
Standing time (mins / 16 h day)	Time spent upright without any stepping. Standardised to 16 h awake and wearing the device.	Standing is stationary light-intensity activity, the least active portion of physical activity. Coupled with sitting and stepping, it forms the complete waking day wearing the activPAL device. Its inclusion is exploratory, and for completeness.
Sitting time (mins / 16 h day)	Total minutes per day spent awake and in a sitting or lying position (sitting/lying). Standardised to 16 h awake and wearing the device.	Excessive sitting time is associated with adverse health outcomes and self-reported sedentary time has previously been linked with walkability.
Mean sitting bout duration (mins)	Total sitting/lying time divided by the number of sitting/lying bouts.	Continuous pattern indicator of the propensity to accumulate sitting in a prolonged uninterrupted manner. Sitting accumulation patterns have shown associations above and beyond sitting time with health outcomes.

All outcome variables exclude time not wearing the device and during time in bed (sleep)

### Potential confounders

We selected the following variables as confounders based on their potential to affect neighbourhood self-selection (i.e., people selecting neighbourhoods to live in that have characteristics that suit their preferred lifestyle), which could influence the exposure and outcome association under investigation [48]. These variables included gender, age, education level (primary and/or secondary school qualification only; trade, technician’s certificate; associate, undergraduate diploma, nursing or teaching qualification; or bachelor’s degree or post-graduate diploma), marital status (married / de facto or not), employment level (full-time employment; part-time employment; self-funded retiree; pension or other benefit; other), income level (no income or not reported, $1–39,999 per year, 40,000–79,999 per year, ≥ 80,000 per year), and children in household (yes or no). Area-level socioeconomic status was also considered as a potential confounder, which was determined using the Index of Relative Socioeconomic Disadvantage (IRSD), a census-based composite indicator of area-level disadvantage [49]. IRSD scores were assigned to the suburbs in which participants resided. In Australia, suburbs represent gazetted localities, typically containing a functional retail area surrounded by residential areas [50]. Participants included in this investigation resided across 209 distinct suburbs.

### Statistical analyses

Statistical analyses were conducted in R software version 4.0 (R Foundation for Statistical Computing, Vienna, Austria). Cohort characteristics and activity behaviour variables were described for the whole sample and compared between low and high walkability neighbourhoods (median split) using analysis of variance for continuous variables and by chi-square tests for categorical variables. All activPAL-derived measures were considered as dependent variables and neighbourhood walkability z-score as the primary independent variable in two-level random intercept models accounting for clustering of participants at the suburb level. With 505 participants across 209 suburbs, we observed clustering at the suburb level was minimal (i.e., low intraclass correlation values). However, given the inclusion of both individual- and area-level variables in our models, we used multilevel modelling as recommended [51]. We used the *lme4* R package to fit models, employing restricted maximum likelihood method for estimation. Dependent variables were converted into z-scores for standardised comparison of effect sizes. Separate two-level linear mixed models included interaction terms for age (< 65; ≥ 65 years old) and sex (male / female) to test the respective influence of age and sex on the associations of walkability with dependent variables. Statistical significance was set at 0.05, without multiple corrections as the study is exploratory.

## Results

Characteristics of the analytical sample are shown in Table 2 with stratification by low and high walkability. The average age of the sample was 59.2 years [range: 36 to 89 years], and 45.7% were women. High walkability neighbourhoods had higher proportions of males, higher educational attainments, and fewer employed, in married or de facto relationships, and in households with children. Higher walkability neighbourhoods were slightly more socioeconomically disadvantaged than were lower walkability neighbourhoods.

**Table 2:** Sample characteristics

		*N*, mean (SD) or %^a^			
		Overall	Low walkability	High walkability	p-value
Sample (n)		505	252	253	
Neighbourhood walkability		0.08 (1.05)	-0.67 (0.34)	0.82 (0.99)	< 0.001
Residential density, counts/km^2^		731.9 (481.6)	440.5 (280.7)	1,022.2 (464.7)	< 0.001
Destinations density, counts/km^2^		1.5 (1.8)	0.5 (0.6)	2.6 (1.9)	< 0.001
Street connectivity - intersection density, counts/km^2^		5.3 (5.8)	1.5 (1.6)	9.1 (6.0)	< 0.001
Age		59.2 (10.6)	58.2 (10.5)	60.1 (10.7)	0.041
Sex (%)	Female	231 (45.7)	119 (47.2)	112 (44.3)	0.564
Education (%)	Primary and/or secondary school qualification only	140 (27.9)	74 (29.6)	66 (26.3)	0.664
	Trade, technician’s certificate	163 (32.5)	82 (32.8)	81 (32.3)	
	Associate, undergraduate diploma, nursing or teaching qualification	73 (14.6)	32 (12.8)	41 (16.3)	
	Bachelor’s degree or higher	125 (25.0)	62 (24.8)	63 (25.1)	
Employment (%)	full-time employment	189 (37.9)	104 (41.9)	85 (33.9)	0.388
	part-time employment	88 (17.6)	44 (17.7)	44 (17.5)	
	Self-funded retiree	83 (16.6)	38 (15.3)	45 (17.9)	
	Pension or other benefit	102 (20.4)	45 (18.1)	57 (22.7)	
	Other	37 (7.4)	17 (6.9)	20 (8.0)	
Income (%)	No income or not reported	31 (6.2)	15 (6.0)	16 (6.4)	0.008
	$1–39,999	116 (23.2)	45 (18.1)	71 (28.3)	
	$40,000–79,999	126 (25.3)	58 (23.4)	68 (27.1)	
	≥$80,000	226 (45.3)	130 (52.4)	96 (38.2)	
Marital Status (%)	Married or de facto	380 (76.2)	206 (83.1)	174 (69.3)	< 0.001
	Not married	119 (23.8)	42 (16.9)	77 (30.7)	
Children in the household (%)	No children	346 (69.3)	165 (66.5)	181 (72.1)	0.21
	Has children	153 (30.7)	83 (33.5)	70 (27.9)	
IRSD at suburb level ^b^ (median [IQR])		1,045 [995, 1,083]	1,063 [1,026, 1,096]	1,020 [980, 1,069]	< 0.001

^a^All values presented as mean (SD) or n and percentage of sample unless stated otherwise

^b^ IRSD (Index of Relative Socioeconomic Disadvantage) corresponds to the suburbs where the participant resided (*n* = 209 suburbs). A score of 1000 represents the national average, and lower scores indicate greater socioeconomic disadvantage

Cohort characteristics were compared between low and high walkability neighbourhoods using t-test for continuous variables and by chi-square tests for categorical variables

Descriptive findings on the activPAL-derived activity measures are shown in Table 3. There were no statistically significant differences in the activity behaviour measures between the low and high walkability strata. Participants spent most of their waking time (87%) in sitting and standing. Most minutes spent stepping in the day were at lower cadences, and in stepping bouts of less than 2 min in duration. The correlations between key variables (walkability and activity pattern measures) are provided in Supplementary Table S1. Highest correlations were observed between sitting and standing (*r* = -0.94), suggesting that breaks from sitting were typically occupied by standing behaviours rather than stepping (sitting vs. stepping *r* = -0.59). Time in purposeful walking bouts was most correlated with mean stepping bout duration (*r* = 0.83) and MVPA (*r* = 0.78), suggesting that more purposeful walking is also typically more intensive in nature. Walkability had modest correlations with outcome variables, with mean stepping cadence (*r* = 0.11), mean stepping duration (*r* = 0.14), purposeful walking minutes (*r* = 0.19) and total MVPA (*r* = 0.14) being statistically significant.

**Table 3:** Activity behaviour variables by low and high walkability

	Overall	Low walkability	High walkability
n	505	252	253
Stepping time (mins / 16 h day)	122.84 (38.42)	124.33 (38.97)	121.36 (37.88)
Mean stepping cadence (steps / min)	77.01 (8.90)	76.76 (8.55)	77.26 (9.25)
Moderate-to-vigorous physical activity (mins / 16 h day)	25.75 (17.49)	25.17 (17.11)	26.33 (17.88)
Mean stepping bout duration (mins)	0.26 (0.06)	0.26 (0.06)	0.26 (0.06)
Purposeful walking time (mins / 16 h day)	22.09 (17.60)	21.19 (17.47)	22.99 (17.71)
Standing time (mins / 16 h day)	296.57 (88.98)	295.54 (86.29)	297.61 (91.74)
Sitting time (mins / 16 h day)	540.58 (106.10)	540.13 (104.91)	541.03 (107.47)
Mean sitting bout duration (mins)	10.90 (3.90)	10.85 (4.15)	10.95 (3.63)

Values presented as mean (SD). No differences between low and high walkability strata for physical activity and pattern variables, *p* > 0.05

**Fig. 1:**
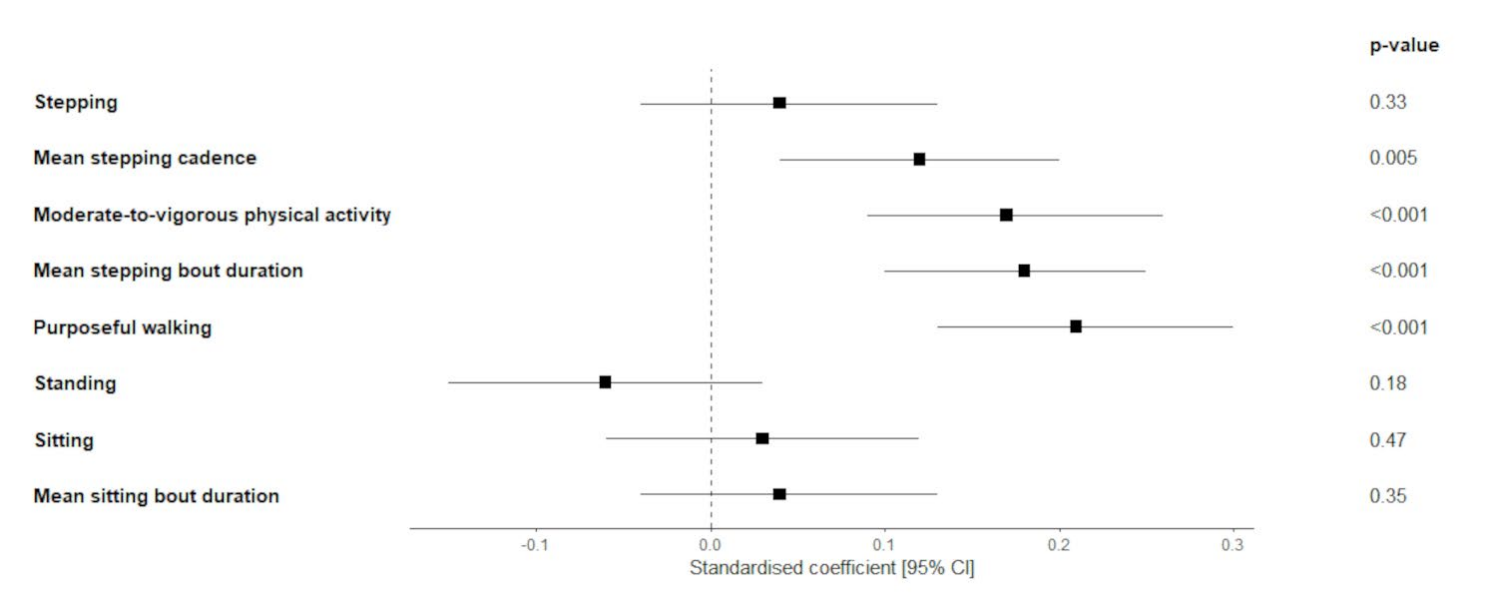
Associations of activPAL derived activity patterns with neighbourhood walkability. Two-level linear regression of neighbourhood walkability with standardized activity and sedentary behaviour variables. Standardized coefficients have all dependent variables converted to z-scores for comparison of effect sizes. Models were used to estimate the associations of neighbourhood walkability with stepping, standing and sitting outcomes, and these models were adjusted for sex, age, education level, employment level, income level, marital status, children in the household, and area-level socioeconomic status accounted for suburb-level clustering. Sample size was 505 participants from major and inner-regional cities of Australia

Figure 1 presents the results of the regression models examining associations of walkability with activity pattern measures. Complete unstandardised and standardised coefficient estimates are shown in Supplementary Table S2. Walkability was not associated with total stepping minutes. However, there were statistically significant positive associations of walkability with mean stepping cadence, total MVPA, mean stepping bout duration, and time in purposeful walking minutes. Walkability was not associated with standing time, sitting time, or mean sitting bout duration.

There were no statistically significant interactions by sex or by age (< 65 years versus ≥ 65 years of age) (Supplementary Table S3 & S4). However, in stratified analyses, middle-aged adults (< 65 years) showed significant positive associations between walkability and certain activity patterns, such as mean stepping cadence, moderate-to-vigorous physical activity, mean stepping bout duration, and purposeful (≥ 2 min) stepping bouts, and these associations were not as strong or significant in the older age group (≥ 65 years). Regarding differences between males and females, associations were similar except for MVPA; males had a non-significant association, while females had a significant positive association.

## Discussion

Present findings add novel evidence on the relations of neighbourhood walkability with residents’ stepping, standing, and sitting patterns. In this cohort of mid-to-older aged Australian adults, neighbourhood walkability was not associated with total daily stepping time, sitting time or standing time. However, positive associations of walkability were found with a subset of stepping patterns; mean stepping cadence, moderate-to-vigorous physical activity, mean stepping bout duration, and time spent in purposeful walking bouts. This set of findings can be interpreted as higher neighbourhood walkability being more supportive of more intensive and longer stepping bouts among residents. Therefore, our findings suggest that people living in higher-walkable areas are more likely to walk briskly and in longer bout durations than those living in lower-walkable areas.

There is a substantial volume of evidence linking neighbourhood walkability and a positive relation with moderate-to-vigorous intensity physical activity [23, 52]. However, there is less evidence on total stepping time [22, 24, 53]. A growing body of evidence suggests that slower cadences, and short stepping bout durations predominantly occur indoors [54] and especially in the home [55, 56]. These patterns of physical activity have high contributions to total stepping time [57, 58]. In contrast, continuous and faster cadences, which are mutually correlated [58], are more likely to occur when someone is transitioning out of their home. Conversely, tasks in the home require routine pauses in ambulation to perform tasks. Whilst not formally investigated in the present study, stepping bouts within the home may be speculated to be usually less than 1 min in their duration [58] and more purposeful stepping, such as walking to a destination, likely accrues in bouts of 2 min or more in duration [59], often above 100 steps/min in cadence in line with the heuristic categorisation of MVPA [60]. Neighbourhoods with higher walkability may enable physical activity patterns associated with leaving the home for errands and active commuting. Notably, whilst more walkable neighbourhoods may promote healthy behaviours such as physical activities that are more continuous and of higher intensity (i.e. brisk walking), our findings suggest less association with total daily stepping time. Whilst higher intensity physical activity is an important modifiable factor for disease prevention, there is a substantial body of evidence indicating that both total stepping time, and light-intensity physical activity are also relevant contributors to health [32, 61]. Previous studies demonstrated that neighbourhood walkability can be positively associated with moderate-to-vigorous physical activity [5]. On the other hand, walkability is often inversely associated with light-intensity activity, with some differences in older cohorts [25]. Together with the current findings, this would suggest higher walkability is linked to higher intensity and more purposeful walking and physical activity, such as that more likely to occur outside of the home such as with commuting, but its association with light-intensity activity may not be constant across subgroups.

Previous research has shown mixed, and often null associations of walkability with total sedentary behaviour [23, 24]. However, examining specific sedentary behaviours has been more informative. In one study of US adults, walkability was unrelated to accelerometer-assessed total sedentary time, but it was associated with lower levels of sedentary time in specific domains such as television watching and driving time [62]. Another study of older adults found that walkability was associated with more instances of sit-to-stand transitions [22], suggesting shorter sitting bouts in more walkable neighbourhoods. However, these findings were not corroborated by present findings on associations with sitting time. While speculative, there may have been a compensatory effect across different domains. For example, high walkable neighbourhoods may support active commuting, which would involve shorter sitting time for transport, but such residents may have longer sitting time at work. This warrants further research, ideally investigating the contexts in which physically active and sedentary behaviours are performed with location-detection devices such as GPS.

The associations of neighbourhood walkability and activity may depend on demographics of age and sex. For example, one study found that neighbourhood walkability was only associated with MVPA in women above the age of 65 [34]. There were similarities with the present study, with only female participants demonstrating significant associations of walkability with moderate-to-vigorous physical activity. In another study, those over the age of 85 showed attenuated associations [22]. Weaker associations with MVPA may be driven by overall lower prevalence of MVPA in older adult populations [34]. This may be partly because older adults are less likely to walk outside of the home [57, 63, 64]. Our findings do not directly corroborate prior studies, but present results do suggest that higher stepping cadences, moderate-to-vigorous physical activity, and longer stepping bout durations may have stronger associations with walkability in people below 65 years of age.

Neighbourhood walkability was most strongly associated with longer and more intensive physical activities in the present study. Existing evidence is suggestive of a hierarchy of behaviour and their associations with health. Specifically, higher intensity activities have the strongest association with health outcomes, and lighter-intensity physical activity and standing have smaller associations, whereas sedentary behaviours have detrimental associations [31]. Given increasing recognition that health outcomes are likely maximised by addressing the continuum of 24-hour movement behaviours [29], further research should investigate how walkable neighbourhoods could facilitate light-intensity physical activity (that contributes significantly to total physical activity) and reduce sedentary behaviour. Further research is particularly needed with older adults, who may stand to benefit the most from modifying these lighter-intensity behaviours [65, 66].

Our findings confirm the supportive role of higher walkability in promoting active travel, which typically involves longer bouts of stepping. However, lack of associations of walkability with total stepping and total sitting time can suggest that those in low walkable neighbourhoods are not necessarily disadvantaged by these behaviour patterns. There are likely to be complex associations among the domains of physical activity and sedentary behaviour, and these may vary by context, including socioeconomic status. For example, high-walkable neighbourhoods may be more likely to be lower-income, and in such cases, residents may have less access to recreation facilities and/or less-safe streets for walking. Though the present study focuses on the “macro-scale” environment features of walkability, there is evidence the “micro-scale” quality of public parks and pedestrian design of streetscapes (e.g., footpaths, street crossings, aesthetics) are more likely to have inequitable distributions [67, 68]. There are multiple strategies for dealing with complex patterns of environmental strengths and weakness. One general approach would be for health care providers and systems to collect assessments of both macro- and micro-scale features using either self-report or GIS-based measures. Then recommendations for increasing physical activity reducing sedentary time could be tailored to each patient’s environmental conditions. This could lead to recommendations to increase physical activity through active travel or at nearby parks and to reduce sitting time by standing while watching TV or working or increasing sit-to-stand transitions at home and/or work. A longer-term approach would be to use assessments of macro- and micro-environment features across many neighbourhoods to determine opportunities for local governments to improve micro-scale features in low-walkable neighbourhoods and correct inequities in activity-supportive microscale features.

Considering that it would be a challenge to promote active travel in low walkable neighbourhoods, encouraging them to replace sitting with stepping in various domains (e.g., at home, at work) may be a potential strategy to reduce cardiovascular risk among those living in low walkable areas. Approaches to implementing physical activity recommendations may benefit from taking into consideration where people reside, as the activity options that they have may be constrained by the environmental characteristics of their neighbourhood environment.

### Strengths and limitations

To our knowledge, this is one of three studies [22, 24] to investigate the relations of neighbourhood walkability and the activPAL thigh-worn monitor and the first to use an objective measure of walkability together with device-derived behavioural patterns in addition to activity and sedentary-time totals. A strength of this study was the device measure used can differentiate sitting from standing and other upright behaviours, and since it is adhered to the thigh it has specificity for capturing leg swinging events indicative of walking [69]. Neighbourhood walkability was objectively measured as a composite index of macroscale environmental attributes (residential, intersection and destination densities) calculated using census and other spatial data. This index has demonstrated longitudinal associations with cardiometabolic health [41]. Future work could investigate the subcomponents of walkability and examine “microscale” features that are relevant to walking, which include directly observed design elements of streetscapes, such as sidewalk presence and quality, safety of street crossings, and aesthetics, including street trees and other greenery. Such features have been associated primarily with walking for transport, but also with other physical activity outcomes, across the life span, even after adjusting for walkability [70]. Another limitation was the relatively small sample (compared to larger survey-measure investigations) that provided device-based measures. The sample was made even smaller by restricting participants to those dwelling in urban areas, where the neighbourhood walkability index has been validated. Considering only urban-dwelling participants limits the generalisability of the study’s findings to remote and rural populations, so further studies are needed to identify associations of relevant built environmental attributes with sedentary behaviour and physical activity patterns in these settings. Although we included commonly used individual sociodemographic variables and area-level socioeconomic status as confounders in our models, there can be still potential residual confounding. For instance, we did not account for neighbourhood self-selection bias, which can be related to both where people live and how they move. While the study was exploratory, notably, conclusions were identical whether applying *p* < 0.05 or a stricter *p* < 0.05/8 significance level to account for the multiple testing across eight regression models. Associations examined were cross-sectional, precluding inferences about whether the built environment could transform peoples’ proclivity for active behaviours. Longitudinal studies are needed to comprehensively understand the relation of built environments to health behaviours, and these investigations should incorporate physical activity pattern metrics (particularly bout length and bout cadence) to understand health impact.

Both the results of the present study and previous literature [22, 62] indicate relations of walkability to the spectrum of activity behaviours could benefit from additional investigations with more nuanced measures of activity behaviours, including the patterns examined in the present study. Of particular relevance, domain-specific device-based measures of physical activity and sedentary behaviour (usually classified as leisure, occupation, transport, and home), which can have distinct associations with built environment attributes [1]. This may be explained by built environments of one environment (e.g., home neighbourhood) being largely irrelevant to behaviour in another setting (e.g., the workplace). On the other hand, an international study with greater environmental and behavioural variability found similar associations of neighbourhood environment variables with self-reported physical activity in leisure and transport domains [71]. This may suggest optimally designed environments have the potential to influence activity behaviours in multiple domains and contribute more strongly to total physical activity and/or sedentary behaviours. Thus, it could be valuable to develop and include device-based measures that can provide additional contextual domain understanding.

## Conclusions

Residents of neighbourhoods with higher walkability had longer duration and higher intensity stepping bouts, which have well-established health benefits. Neighbourhood environments may have a greater influence on the patterns of activity behaviour, rather than on the total volume of activity. This may be a key mechanism through which the built environment affects health. Future research needs to investigate whether the patterns of physical activity are an underlying pathway between walkability and health outcomes.

## Electronic supplementary material

Below is the link to the electronic supplementary material.

Supplementary Material 1

Supplementary Material 2

## Data Availability

Datasets are available on request to corresponding author.

## Abbreviations

AusDiab: Australian Diabetes, Obesity and Lifestyle Study
IRSD: Index of Relative Socioeconomic Disadvantage
MVPA: Moderate-to-vigorous physical activity
