# Supplementary material for: Associations of neighbourhood walkability with patterns of device-measured stepping, standing and sitting
Source: Int J Behav Nutr Phys Act. 2025 Apr 9;22:41. doi: 10.1186/s12966-025-01737-4 (PMC11983810; doi:10.1186/s12966-025-01737-4)
Supplement: Supplementary file 2 — Supplementary Material 2 [file 12966_2025_1737_MOESM2_ESM.docx]

**Electronic Supplementary Material**

*Table S1: Correlations between key variables*

|  | 1 | 2 | 3 | 4 | 5 | 6 | 7 | 8 | 9 |
| --- | --- | --- | --- | --- | --- | --- | --- | --- | --- |
| 1. Walkability index | — |  |  |  |  |  |  |  |  |
| 2. Stepping minutes | 0.00 | — |  |  |  |  |  |  |  |
| 3. Mean stepping cadence | 0.11* | 0.20* | — |  |  |  |  |  |  |
| 4. Moderate-to-vigorous stepping time | 0.14* | 0.58* | 0.66* | — |  |  |  |  |  |
| 5. Mean stepping bout duration | 0.14* | 0.44* | 0.59* | 0.63* | — |  |  |  |  |
| 6. ≥2 min stepping bouts | 0.19* | 0.55* | 0.53* | 0.78* | 0.83* | — |  |  |  |
| 7. Standing minutes | -0.05 | 0.27* | -0.22* | -0.04 | -0.46* | -0.19* | — |  |  |
| 8. Sitting minutes | 0.04 | -0.59* | 0.11* | -0.18* | 0.23* | -0.04 | -0.94* | — |  |
| 9. Mean sitting bout duration | 0.04 | -0.54* | -0.15* | -0.24* | 0.07 | -0.08 | -0.45* | 0.58* | — |
| * *p*-value <0.05  Correlations were derived using Pearson method.  n = 505 | | | | | | | | | |

*Table S2. Associations of activPAL derived activity patterns with neighbourhood walkability*

|  | Unstandardised coefficients (n=505) | Standardised coefficients (n=505) | |
| --- | --- | --- | --- |
|  | B [95% CI] | β [95% CI] | p-value |
| Stepping | 1.69 [-1.66, 5.02] | 0.04 [-0.04, 0.13] | 0.332 |
| Mean stepping cadence, *steps/min* | 1.06 [0.33, 1.79] | 0.12 [0.04, 0.2] | 0.005 |
| Moderate-to-vigorous stepping time | 2.99 [1.53, 4.46] | 0.17 [0.09, 0.26] | <0.001 |
| Mean stepping bout duration | 0.01 [0.01, 0.02] | 0.18 [0.1, 0.25] | <0.001 |
| ≥2 min stepping bouts | 3.76 [2.28, 5.23] | 0.21 [0.13, 0.3] | <0.001 |
| Standing | -5.35 [-13.06, 2.31] | -0.06 [-0.15, 0.03] | 0.18 |
| Sitting | 3.48 [-5.88, 12.88] | 0.03 [-0.06, 0.12] | 0.474 |
| Mean sitting bout duration | 0.16 [-0.17, 0.5] | 0.04 [-0.04, 0.13] | 0.351 |
| Two-level linear regression of neighbourhood walkability with unstandardised and standardised activity and sedentary behaviour variables.  Unstandardised coefficients represent number of minutes (unless indicated otherwise) associated with 1-unit of neighbourhood walkability z-score.  Standardised coefficients have all dependent variables converted to z-scores for comparison of effect sizes.  Models were adjusted for sex, age, education level, employment level, income level, marital status, children in the household, and area-level socioeconomic status accounted for suburb-level clustering. | | | |

*Table S3: Association of walkability with activity and sedentary behaviour patterns by sex*

|  | Stratified unstandardized coefficients b [95% CI] | | |
| --- | --- | --- | --- |
|  | Male (n=274) | Female (n=231) | Interaction *p*-value^a^ |
| Stepping | 3.39 [-2.32, 9.08] | 1.05 [-2.94, 5.06] | 0.315 |
| Mean stepping cadence, *steps/min* | 1.24 [0.00, 2.48] | 0.97 [0.07, 1.87] | 0.502 |
| Moderate-to-vigorous stepping time | 2.14 [-0.59, 4.89] | 3.47 [1.71, 5.13] | 0.674 |
| Mean stepping bout duration | 0.01 [0, 0.02] | 0.01 [0, 0.01] | 0.244 |
| ≥2 min stepping bouts | 4.48 [1.81, 7.15] | 3.68 [1.9, 5.42] | 0.367 |
| Standing | -3.38 [-19.02, 12.32] | 6.68 [-4.87, 18.25] | 0.384 |
| Sitting | 0.17 [-0.49, 0.84] | 0.17 [-0.15, 0.50] | 0.265 |
| Mean sitting bout duration | 0.8 [-12.01, 13.66] | -7.82 [-17.45, 1.82] | 0.721 |
| Two-level linear regression of neighbourhood walkability with unstandardised and standardised activity and sedentary behaviour variables.  stratified by sex.  Unstandardised coefficients represent number of minutes (unless indicated otherwise) associated with 1-unit of neighbourhood walkability z-score.  Models were adjusted for sex, age, education level, employment level, income level, marital status, children in the household, and area-level socioeconomic status accounted for suburb-level clustering.  ^a^ Sex variable added to the overall model (n=505) as an interaction term to test whether the associations with neighbourhood walkability were significantly different by sex. | | | |

*Table S4: Association of walkability with activity and sedentary behaviour patterns by age group*

|  | Stratified unstandardized coefficients b [95% CI] | |  |
| --- | --- | --- | --- |
|  | <65 years (n=349) | ≥65 years (n=156) | Interaction *p*-value^a^ |
| Stepping | 1.95 [-1.77, 5.65] | -0.08 [-8.12, 7.96] | 0.949 |
| Mean stepping cadence, *steps/min* | 1.34 [0.53, 2.16] | -0.33 [-2.18, 1.52] | 0.258 |
| Moderate-to-vigorous stepping time | 3.55 [1.92, 5.24] | 0.49 [-2.83, 3.81] | 0.200 |
| Mean stepping bout duration | 0.01 [0.01, 0.02] | 0.00 [-0.01, 0.02] | 0.236 |
| ≥2 min stepping bouts | 4.35 [2.69, 6.02] | 0.78 [-2.64, 4.21] | 0.138 |
| Standing | 2.79 [-7.42, 13.1] | 11.26 [-12.09, 33.93] | 0.612 |
| Sitting | 0.14 [-0.17, 0.46] | 0.28 [-0.74, 1.3] | 0.659 |
| Mean sitting bout duration | -5.15 [-13.7, 3.27] | -11.77 [-30.08, 7.04] | 0.452 |
| Two-level linear regression of neighbourhood walkability with unstandardised and standardised activity and sedentary behaviour variables.  stratified by age category.  Unstandardised coefficients represent number of minutes (unless indicated otherwise) associated with 1-unit of neighbourhood walkability z-score.  Models were adjusted for sex, age, education level, employment level, income level, marital status, children in the household, and area-level socioeconomic status accounted for suburb-level clustering.  ^a^ Age category variable added to the overall model (n=505) as an interaction term to test whether the associations with neighbourhood walkability were significantly different by age category. | | | |
